# Hygiene of the Teeth

**Published:** 1898-11

**Authors:** 


### Hygiene of the Teeth.

In the *Sanitarian* for July for 1898, Dr. Clement B. Lowe, writing on the care of the teeth, thinks that we do not value them sufficiently until we have lost them. He quotes Lauder Brunton as authority for the statement that the increase in the average length of life is largely due to the fact that the dentists supply teeth to those who would not otherwise have them, so that people can continue to masticate their food for a very much longer time than before.

Some of our poor teeth may be directly chargeable to the character of the flour, which by fine grinding and repeated bolting has been deprived of much of its gluten and phosphates, and consists largely of fine starch.

The eruption of the teeth is a critical period. At this time the tooth has not reached its full development, and the fang has a sharp edge which, by the downward pressure of the resisting gum, presses the nerve against the bony process of the jaw-bone, causing much pain. Lancing often gives instant relief. If the tooth does not immediately erupt, the resultant cicatrix upon the mucous membrane is, contrary to general belief, less resistant than the original tissue.

A quill toothpick should be used after each meal, and the mouth thoroughly rinsed; and a brush and alkaline tooth-powder used before retiring. Tooth-powders containing gritty substances are to be condemned, as also strongly alkaline soaps and tooth-washes. Acids and any considerable quantities of astringents are also inadvisable. Prepared chalk is the best base.

No tooth should be extracted if it is possible to save it. Not only is it lost, but its opponent has nothing to grind against, and is useless.—*Medicine.*

